# Eicosapentaenoic acid loaded silica nanoemulsion attenuates hepatic inflammation through the enhancement of cell membrane components

**DOI:** 10.1186/s12575-022-00173-z

**Published:** 2022-09-07

**Authors:** Jihan Hussein, Mona A. El-Bana, Zakaria El-kHayat, Mehrez E. El-Naggar, Abdel Razik Farrag, Dalia Medhat

**Affiliations:** 1 Medical Biochemistry Department, National Research Center, 33 El Behouth St.Dokki, Giza, 12622 Egypt; 2 Pre-Treatment and Finishing of Cellulosic Fabric Department, National Research Centre, Dokki, Giza, Egypt; 3 Pathology Department, National Research Centre, Dokki, Giza, Egypt

**Keywords:** Diethyl nitrosamine, 8-hydroxyguanozine, Arachidonic acid, Alpha-Linolenic acid, HPLC

## Abstract

**Background:**

Liver inflammation is a multistep process that is linked with cell membrane fatty acids composition. The effectiveness of eicosapentaenoic acid (EPA) undergoes an irreversible change during processing due to their unsaturated nature; so the formation of nanocarrier for EPA is crucial for improving EPA’s bioavailability and pharmacological properties.

**Objective:**

In this study we aimed to evaluate the efficiency of EPA alone or loaded silica nanoemulsion on the management of hepatic inflammation induced by diethyl nitrosamine (DEN) through the enhancement of the cell membrane structure and functions.

**Methods:**

The new formula of EPA was prepared to modify the properties of EPA. Forty-eight male Wistar albino rats were classified into: control, EPA, EPA loaded silica nanoemulsion (EPA–NE), DEN induced hepatic inflammation; DEN induced hepatic inflammation treated with EPA or EPA –NE groups. Plasma tumor necrosis factor alpha (TNF-α), interleukin-1 beta (IL-1β), liver hydroxyproline (Hyp) content, and liver oxidant and anti-oxidants were estimated. Urinary 8- hydroxyguanozine (8- OHdG) and erythrocyte membrane fatty acids fractions were estimated by High-performance liquid chromatography (HPLC). Also, histopathology studies were done to verify our hypothesis.

**Results:**

It was appeared that administration of EPA, in particular EPA loaded silica nanoemulsion, ameliorated the inflammatory response, increased the activity of the anti-oxidants, reduced levels of oxidants, and improved cell membrane structure compared to hepatic inflammation induced by DEN group. Histopathological examination confirmed these results.

**Conclusion:**

EPA and notably EPA loaded silica nanoemulsion strongly recommended as a promising supplement in the management of hepatic inflammation.

## Introduction

The liver is the largest organ in the body responsible for nutrient metabolism and protein synthesis [1], it also involved in biotransformation of food and drugs [2]. Liver cancer is considered one of the most common causes of cancer- related death [3].

Liver carcinoma is a multistep process including numerous risk factors that promote damaging of genes as well as molecular and cellular deregulations and hepatocytes transformation [4].

Hepatocellular carcinoma (HCC) represents more than 90% of liver cancers and constitutes a major worldwide health problem. HCC is the ending process of chronic liver diseases, starting from fibrosis and cirrhosis [5].

Numerous investigations have indicated that the most common causative incidents in liver diseases are oxidative stress and inflammation. An uncontrolled and prolonged imbalance between the generation of free radicals and their removal by defensive mechanisms causes serious damage to cells, with potentially consequences for the entire organism, resulting in a wide range of chronic diseases [6–8]. During liver damage, ROS can trigger the expression of pro-inflammatory genes, which is a risk factor for liver diseases [6].

The influx of neutrophils, monocytes, and lymphocytes, as inflammatory cells, to the stimulation’ site is a fundamental component of inflammation. The activated inflammatory cells at the site of inflammation release chemical mediators such as chemokines, cytokines, eicosanoids and nitric oxide causing elevation in superoxide, hydroxyl radical, hydrogen peroxide and tissue damage [6, 9, 10]. Thus, the over expression of the pro-inflammatory genes activates an intracellular signaling cascade generating additional ROS which in turn increase the oxidative stress and inflammatory lesion which drive the progression of liver disorders [6].

Most therapeutics of liver disorders is designed to interact with the proteins and nucleic acids also alteration in the cell membrane lipid composition is associated in functionality of cancer cells. Treatment with Polyunsaturated fatty acids (PUFA) is becoming a very exciting alternative, above all omega -3 fatty acids have been shown to exert a number of beneficial biological properties, including anti-cancer effects; supplementation of n-3 polyunsaturated fatty acids (PUFA) has inverse association with the risk of HCC [11].

Eicosapentaenoic acid (EPA), an omega-3 polyunsaturated fatty acid can modify cell membrane configuration as well as equilibrium between ceramide and sphinomyelin and hence changing its properties like permeability and fluidity [12]*.* In addition, it has inhibitory effect on the growth of tumor cells with little or no cytotoxic effect on the normal cells [11].

EPA is commonly found in fish oil, and its effectiveness undergoes an irreversible change during processing due to their unsaturated nature; so the formation of nanocarrier for EPA is crucial for improving EPA’s bioavailability and pharmacological properties, and widening its use in biomedical fields.

### Aim of the work

From this point of view, this study aimed to evaluate the efficiency of EPA alone and EPA loaded silica nanoemulsion on the management of hepatic inflammation induced by Diethyl nitrosamine (DEN) in experimental rats via the modulation of the erythrocyte membrane composition.

## Materials and methods

### Materials

#### Chemicals

Tetraethyl orthosilicate (TEOS) was purchased from Fischer Co. (Germany). Tween 80 and chemphore were purchased from Across Co. (Germany).

Eicosapentaenoic acid, Diethyl nitrosamine (DEN), HPLC standards for 8- hydroxyguanozine and fatty acid fractions were purchased from Sigma Chemical Company, St. Louis, MO, USA. All using chemicals were HPLC grade.

### Methods

#### Preparation of Eicosapentaenoic acid (EPA) nanoemulsion

To prepare EPA in nanoemulsion form, tetraethyl orthosilicate (TEOS; 5 mL) was dissolved in water for 5 min. at room temperature using magnetic stirring. After that chemphore (4 mL /25 mL H_2_O) containing 15 mL of EPA were added to TEOS solution and kept under vigorous stirring using ultrasonic homogenizer (15 min). At the end of homogenization, the colorless solution was turned to milky solution confirming the emulsification of EPA.

#### Characterization

TEM images of EPA loaded silica nanoemulsion were acquired by JEM-2200-FS Field emission transmission electron microscope (JEOL, Japan) to an operating voltage of 200 kV. The grids were given hydrophilic treatment using Joel Datum HDT-400 hydrophilic treatment device. TEM samples were prepared by dropping the diluted nanoemulsion into the carbon coated hydrophilic copper TEM grids.

The particle size distribution and  polydispersity index of EPA loaded silica nanoemulsion (EPA-NE) were determined in triplicates by a photon correlation spectroscopy (PCS) using a zetasizer (Malvern Zetasizer Nano ZS90, UK). Approximately, 1 mL of EPA loaded silica nanoemulsion were diluted with 1 mL of deionized water. The dissolved samples were sonicated for 30 min in ice bath. The samples were placed in a zetasizer and the particle size, and polydispersity index were then observed.

#### Experimental design

##### Animals

Male Wistar albino rats weighting 150 ± 10 g were obtained from the animal house of National Research Centre (NRC), Giza, Egypt. The animals were housed in stainless steel cages at the temperature range of 22 + 2 °C, under a 12-h light/12-h dark cycle, and allowed to acclimatize for a period of 10 days to the experiment. The whole experiment was approved by the ethical committee of NRC (Ethical approval number 19 212).

##### Induction of hepatic inflammation

DEN was dissolved in 0.9% normal saline, rats were induced with a single injection of DEN in a dose of 1 mg /kg b.wt. by intraperitoneal injection (i.p.) [13].

#### Experimental design

Forty-eight male Wistar albino rats were classified into six groups as follow (8 rats in each group):

- Negative control group: rats were orally administered with 0.5 ml vehicle solution (0.9% normal saline).
- EPA group: rats were administered with EPA (500 mg/kg b.wt.) per day orally for 4 weeks [14].
- EPA loaded silica nanoemulsion group (EPA-NE): rats were administered with EPA loaded silica nanoemulsion (500 mg/kg b.wt.) per day orally for 4 weeks.
- DEN induced hepatic inflammation group: rats were treated with diethyl nitrosamine (DEN) (1 mg/kg b.wt.) in a single dose [13].
- DEN induced hepatic inflammation and treated with EPA group (Treated I): rats were treated with DEN (1 mg /kg b.wt.) in a single dose and in the same time treated with EPA (500 mg/kg b.wt.) per day orally for 4 weeks.
- DEN induced hepatic inflammation and treated with EPA –NE group (Treated II): rats were treated with DEN (1 mg /kg b.wt.) in a single dose and in the same time treated with EPA loaded silica nanoemulsion (500 mg/kg b.wt.) per day orally for 4 weeks.
- After the experimental period; 24 h urine samples were collected from each animal using metabolic cages for determination of urinary 8- hydroxyguanozine. Rats were fasted for twelve hours and the blood was withdrawn from the optical vein. The blood samples were collected with EDTA to isolate erythrocyte membrane as described previously [15] and the separated plasma was used for biochemical parameters estimations.
- Liver was discarded quickly from each rat and washed with ice-cold saline. The first part of the liver was homogenized in 0.1 M Tris buffer for biochemical estimations. The second part was used for histopathological study.

### Biochemical analysis

#### Determination of liver functions

Plasma Alanine Aminotransferase (ALT) and aspartate aminotransferase (AST) activities were estimated by colorimetric method [16]*.* Also, albumin and total protein were determined colorimetrically [17].

#### Determination of liver oxidant /antioxidant parameters

Liver thiobarbituric acid-reactive substances (TBARS), superoxide dismutase (SOD) and reduced glutathione (GSH) were determined colorimetrically [18–20] respectively using commercial kits from Biodiagnostics, Egypt.

#### Determination of plasma inflammatory markers

Plasma tumor necrosis factor alpha (TNF-α) and interlukin – 1 beta (IL-1β) were estimated by enzyme linked immunosorbent assay using ELISA kit according to manufacture procedures.

#### Determination of liver hydroxyproline content (Hyp)

To estimate hydroxyproline content, a colorimetric experiment was done using the Patiyal and Katoch technique. Briefly, liver Sects. (0.5 g) were hydrolyzed (20 h in 6 mol/L HCl at 100 °C), diluted in ultrapure water, and centrifuged to eliminate contaminants. At room temperature, samples were incubated for 10 min in 0.05 mol/L chloramine-T (Fisher, Fair Lawn, NJ, USA), followed by a 15-min incubation in Ehrlich’sperchloric acid solution at 65 °C. At 561 nm, sample absorbance was measured, and the value for each sample was computed using a hydroxyproline standard curve [21, 22].

#### Determination of erythrocyte membrane fatty acids fractions

Erythrocyte membrane fatty acids fractions including EPA, arachidonic acid (AA), linoleic acid (LA), and alpha linolenic acid (ALA) were estimated by high performance liquid chromatography (HPLC). The cell membrane was homogenized in a 2% acetic acid / diethyl ether combination (2:1 volume ratio). After filtering and centrifuging the solution at 500 xg, the organic phase was evaporated to dryness. The extract was dissolved in acetonitrile (200 µL).

##### HPLC condition

The HPLC system (Agilent technologies 1100 series) was equipped with a quaternary pump (Quat Pump, G131A model) and the C18 column (260 X 4.6, particle size 5 µm). As the mobile phase, an acetonitrile/water (70/30) v/v mixture was utilized, and it was provided by isocratic elution at a flow rate of 1 ml/min and a wave length of 200 nm. The peak areas of standards were determined after repeated dilutions were injected. By graphing peak areas vs. concentrations, a linear standard curve was drowning. The standard curve was used to calculate the concentration of the samples [23].

#### Determination of urinary 8- hydroxyguanozine (8- OHdG)

Rats were fasted and placed in metabolic cages for 24 h to collect urine. Urine samples were stored at—20 °C until analyzed. The concentration of 8-OHdG was determined using HPLC system [24, 25].

In brief, the 8-OHdG standard was dissolved in ultrapure water, and then successive dilutions were made and injected onto HPLC to create a standard curve with varying concentrations.

##### Processing of samples

Strata C18-E (55 um, 70A) column was used to extract 8-OHdG from a 1 ml urine sample. The eluents were dried with a nitrogen gas stream and reconstituted in 5 mL of ultrapure water. HPLC was injected with 20 µL of each sample.

##### HPLC  condition

The mobile phase is a 25/10/965 v/v mixture of acetonitrile, methanol, and phosphate buffer. Phosphate buffer was prepared by dissolving 8.8 g of potassium dihydrogen phosphate (KH_2_PO_4_) in 1000 ml ultrapure water and adjusting the pH to 3.5.The buffer was then filtered twice using a 0.45 m pore size sterile membrane filter before passing over an HPLC reverse phase column and electrochemical detector with a cell potential of 600 mV at a flow rate of 1 ml/min.

The urine 8-OHdG concentration was derived from the standard curve and divided by the urinary creatinine, which was obtained using Larsen’s kinetic technique [26].

### Liver histopathological examination

At the end of the experiment, sections from the liver were fixed in 10% buffered formalin for 24 h, and then dehydrated with grades of ethanol (70%, 80%, 90%, 95% and 100%). Dehydration was then followed by clearing the samples in two changes of xylene. Samples were impregnated with two changes of molten paraffin wax and embedded and blocked out. Sections (5 µm) were stained [27–29] Oxidative stress: molecular perception and transduction of signalswith the following conventional histological stain: hematoxylin and eosin routinely processed.

### Statistical analysis

SPSS 13.0 statistical software was used to examine the data (SPSS, Chicago, IL). Data were analysed using repeated-measures one way ANOVA. A statistically significant probability was defined as one with a probability of less than 5% (P 0.05).

## Results and discussion

Encapsulation of EPA into silica nanoemulsion was carried out using water/oil nanoemulsion. Emulsifying agent such as Tween 80 and chemphore were used to further disperse the formed nanoemulsion. Thus, our work is to utilize the pores of silica nanoemulsion as a holder for EPA drug. The porous silica nanoemulsion was prepared via using TEOS as precursor and chemphore as surfactant to kep the particles of the formed silica nanoemulsion a way from each others with the aid of ultrasonic homogenizer that achieve the full dispersition for the formed nanoemulsion with there is no phase separation for the components of the resultant nanoemulsion.

**Fig. 1:**
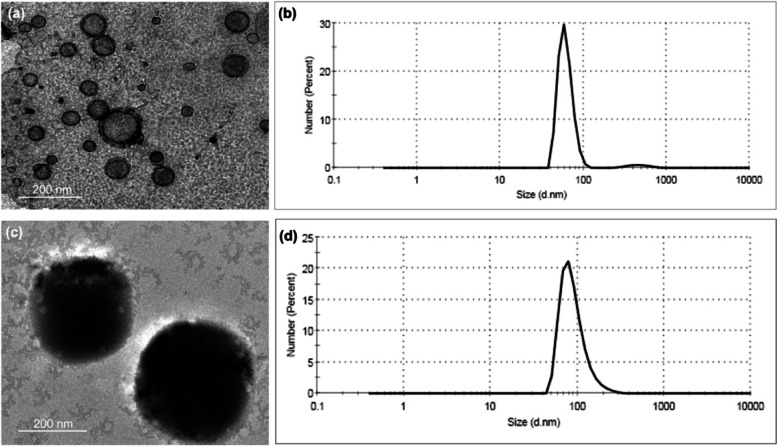
TEM, average particle size of (**a**, **b**) silica nanoemulsion and (**c**, **d**) EPA loaded silica nanoemulsion

As clearly remarkabe from Fig. 1a that the silica nanoemulsion are prepared with porosity and formed as hollow spherical particles. The DLS of silica nanoemulsion (Fig. 1b) displayed that the particles are phormed with very small size (61.58 nm) with PdI = 0.05. As known from the value of PdI, it can be concluded that the particles are formed with monodisperes particles with no noticable for the agglomeration. In comparison with EPA encapsulated silica nanoemulsion, the particles are filled with EPA. Via eye visullization, silica nanoemulsion (Fig. 1a), the spherical particles are appeared as porous particles, these porosity are appeared as black particles while encapsulation of EPA (Fig. 1c). The average particle size analyzer for EPA loaded silica nanoemulsion (Fig. 1d) is increased to 90 nm with pdI equal to 0.074. The particle size was enlarged due to the encapsulation of EPA. Overall, the hydrodymanimc average size is around 100 nm which is not significantly affected on the efficiency of EPA. Additionally, there is a difference for the diameter size for the produced nanoemuslion when evalauted via TEM and DLS which is mainly attributed to the difference in the utilized technique.

DEN is a secondary alkylating agent which generates ROS leading to an oxidation of DNA and/or RNA molecules. It is generally found in smoked and fried food items. It is a confirmed hepatotoxic in rodent models [30].

ALT and AST, the liver biomarker enzymes, are suggestive of the beginning of hepatocellular injury. These enzymes are existed in the cytoplas and liberated into the blood due [31].

**Fig. 2:**
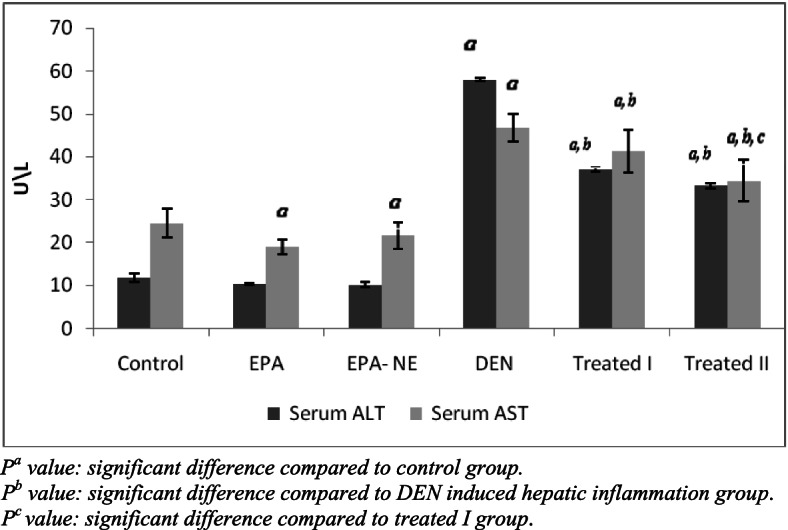
Levels of serum ALT and AST in different studied groups. *P*^*a*^ value: significant difference compared to control group. *P*^b^ value: significant difference compared to DEN induced hepatic inflammation group. *P*^*c*^ value: significant difference compared to treated I group

It was found that the rats administered DEN significantly increased levels of serum ALT and AST (Fig. 2).

Excessive generation of free radicals or depletion of antioxidant enzymes cause oxidative damage by disrupting the cellular redox balance between the production of oxidants and antioxidant defense [32].

**Fig. 3:**
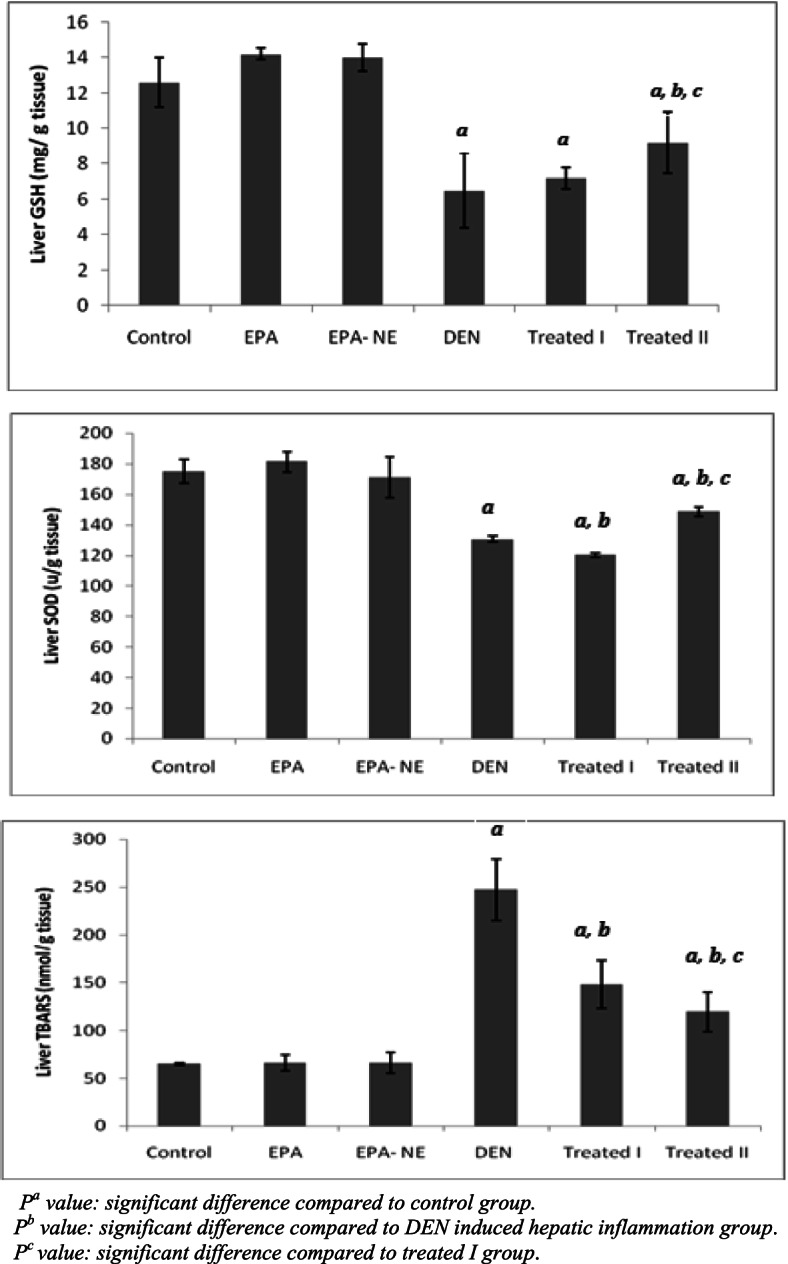
Liver oxidant / antioxidant markers in different studied groups. *P*^*a*^ value: significant difference compared to control group. *P*^*b*^ value: significant difference compared to DEN induced hepatic inflammation group. *P*^*c*^ value: significant difference compared to treated I group

The parenchymal cells of the liver are the most vulnerable cells to oxidative stress [33], which is owing to the capacity of certain organelles found inside parenchymal cells (mitochondria, microsomes and peroxisomes) to produce free radicals that can cause fatty acid oxidation, making the liver a key target for ROS damage [34]. Oxidations of biological macromolecules especially DNA, proteins, and lipids are considered the most trademark oxidative stress induced by DEN [35]. Level of  liver GSH,    activity of antioxidant enzyme     SOD, and level of TBARS were measured during this investigation, and the results revealed that administration of DEN significantly elevated the extent of lipid peroxidation, as shown by the elevation of TBARS and decreased level of GSH, and SOD activity (Fig. 3).

 SOD and GSH are the first line of antioxidant defense system in cells; they protect the cells by scavenging free radicals, altering them to less toxic metabolites [36]. Results from this study showed a reduction in the concentration of GSH and activity of SOD enzyme after administered of DEN in hepatic inflammation group.

These findings are consistent with the observations of Adebayo and his colleagues who reported the increase in the levels of TBARS with a concomitant decrease in antioxidant defense system in the DEN-administered mice [31].

In the present investigation, the supplementations of EPA especially EPA loaded silica nanoemulsion ameliorated activities of serum ALT and AST, enhanced the anti-oxidative status (GSH, SOD), and blunted the oxidative stress level (TBARS) in the treated groups (Figs. 2 and 3). In support, in animal models of liver injury evoked by drugs, alcohol or other, EPA terminated oxidative stress, and mitochondrial dysfunction [37, 38].

Further, EPA was described to motivate reactions that regulate the expression of detoxifying/ antioxidant genes and obstruct inflammation [39]. EPA reacts directly with the negative regulator of Nrf2, Keap1, and initiates dissociation of Keap1 with Cullin 3, thereby inducing Nrf2-directed antioxidant gene expression [40, 41].

Moreover, Tanaka et al. found that EPA administration to mice significantly reduces hepatic TBARS through enhanced expression of zinc, copper, and manganese-SOD [42].

**Fig. 4:**
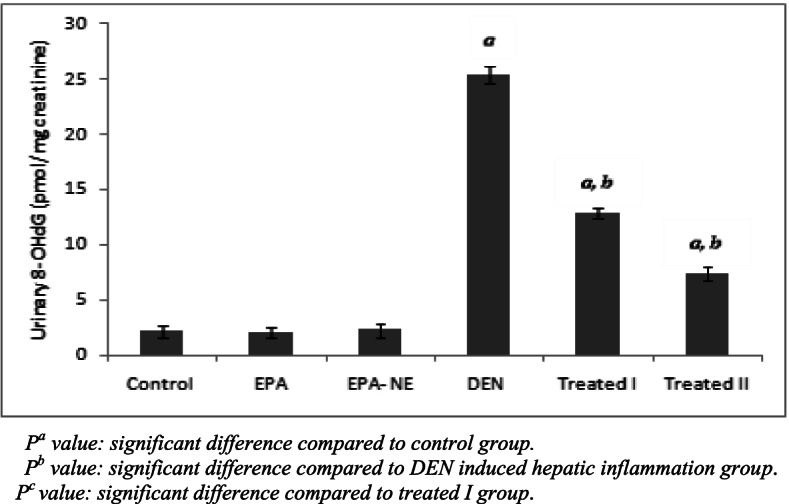
Urinary 8- hydroxyguanozine levels in different studied groups. *P*^*a*^ value: significant difference compared to control group. *P*^*b*^ value: significant difference compared to DEN induced hepatic inflammation group. *P*^*c*^ value: significant difference compared to treated I group

Oxidative DNA damage is associated with hepatocarcinogenesis development. Over production free radicals such as ROS is considered an important factor in genetic instability during liver inflammation [43]. 8-OHdG is another significant biomarker of ROS-induced oxidative DNA damage, as well recognized a risk factor for hepatocellular carcinoma in patients with chronic liver inflammation [44]. In this study, the 8-OHdG level in urine of DEN-administered rats was significantly elevated as compared with the control group (Fig. 4). Consistent with this finding, recent studies reported that cigarette smoke, which is rich in DEN, causes an accumulation of 8-OHdG in the lungs as a result of increases oxygen free radicals, leading to inflammatory responses, fibrosis, and tumor growth [45, 46].

On the other hand, we found that EPA supplementations particularly EPA loaded silica nanoemulsion reduced the level of urinary 8-OHdG urine levels compared to DEN induced hepatic inflammation group (Fig. 4); which confirmed the ability of EPA to counteract oxidative modification of DNA. Previous study demonstrated that fish oil rich with EPA were significantly decreased the levels of oxidative DNA damage (8-OHdG) in male cigarette smokers and attributed that the beneficial effect of EPA on suppressing the generation of reactive oxygen species [47]. Furthermore, dietary fish oil protects against colon cancer in rats by reducing oxidative DNA damage, as measured by a quantitative immunohistochemistry study of 8-OHdG [48].

**Fig. 5:**
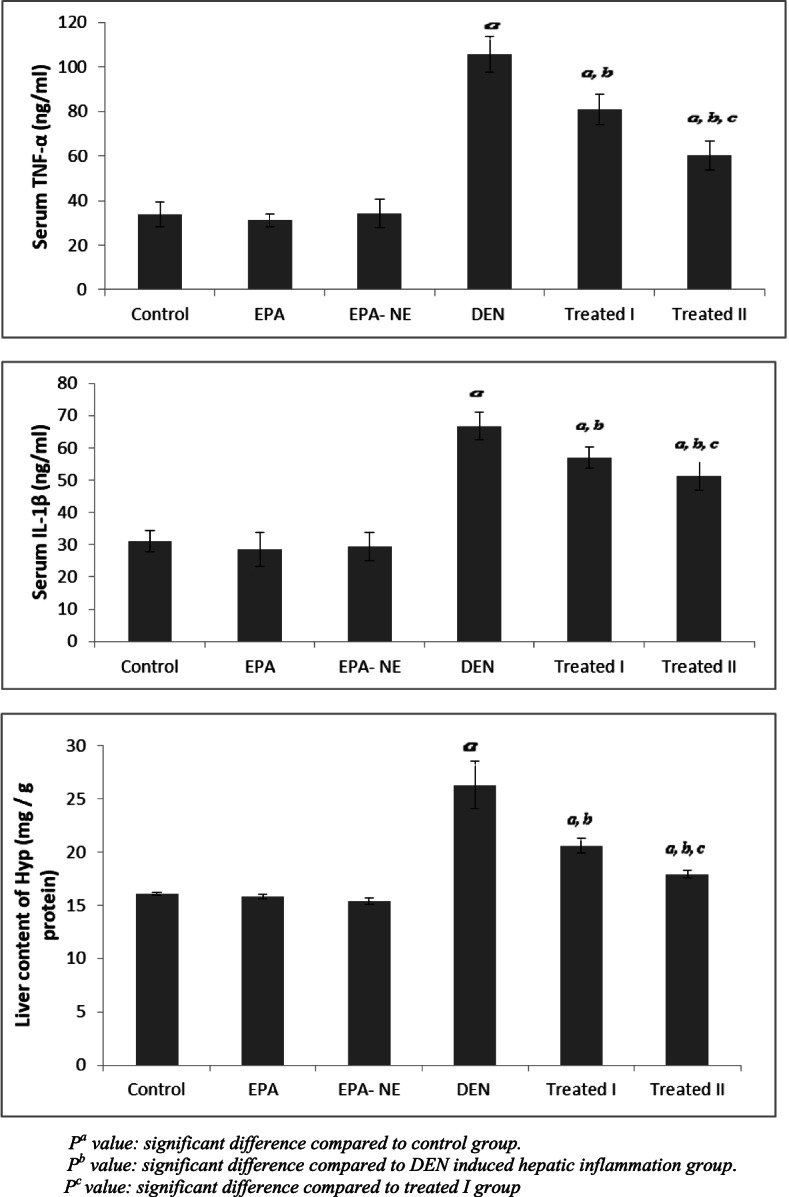
Serum Inflammatory markers and liver hydroxyproline content in different studied groups. *P*^*a*^ value: significant difference compared to control group. *P*^*b*^ value: significant difference compared to DEN induced hepatic inflammation group. *P*^*c*^ value: significant difference compared to treated I group

Chronic inflammation of the liver is a well-recognized risk factor for carcinogenesis, the molecular link between inflammation, hepatic fibrogenesis, and hepatocellular carcinoma [49]. Regarding DEN-induced hepatic inflammation group, concentrations of TNF-α, IL-1b were significantly elevated (Fig. 5). Consistent with our study, Ding et al. found that TNF-α, IL-1b where up-regulated during DEN-exposed, causing hepatic inflammation [50]. These results were due to the de-alkylation of DEN to its active mutagenic metabolites which modulated substances such as 3-methylcholanthrene and phenobarbital (PB) which in turn increase hepatic demethylase activity. Besides, oxidative stress induced by DEN is well documented as a factor to the pathogenesis of hepatic carcinogenesis [51].

DEN activates the myeloid differentiation primary response 88 (MyD88) dependent and MyD88-independent signaling cascades [52]. The MyD88-dependent signal transduction activates NF-kB through activation of its inhibitory protein IkBa, which allows NF-kB nuclear translocation and controls the expression of a multitude of pro-inflammatory cytokines and other immune-related genes, such as TNF-a, IL-1, IL-1β, IL-6, and IL-12 [49].

Liver inflammation is a hallmark of early-stage fibrosis, which can extend to severe fibrosis and cirrhosis [53]. Fibrosis is characterized by the accumulation of collagen and other extracellular matrix components[54]. One of the main approaches for fibrosis quantification and also for the therapeutic assessment of new anti-fibrotic drugs is to determine the hydroxyproline (Hyp) level of the liver [55]. In the current study, increased production of fibrillary collagens was confirmed by a significantly increased hydroxyproline level in the DEN- administered group (Fig. 5). A number of studies have also shown the fast buildup of collagen during nitrosamine-induced liver fibrosis [56, 57].

Interestingly, our results showed less severe inflammatory response in groups administered EPA essentially EPA loaded silica nanoemulsion reported by the reduction in serum levels of TNF-α, IL-1b compared to DEN induced hepatic inflammation group (Fig. 5). Our findings are following Albracht-Schulte et al. who reported that EPA exhibits protective effects in liver steatosis and inflammation through decreased NF-kB and pro-inflammatory cytokines, such as TNF-α and Mcp-1, as well as increases in anti-inflammatory cytokines, such as IL-10 via up-regulation of miR-let-7 and inhibition of MAPK/ERK/JNK pathways. Furthermore EPA can well incorporate into the liver, and suppress gene expression of the pro-inflammatory cytokines, IL-1b, IL-6 and interferon-γ [37].

We have reported in this study that EPA, considerably EPA loaded silica nanoemulsion represses DEN-induced nodule formation and suppresses sub-sequential fibrosis which is demonstrated through decreased hepatic Hyp content in the treated groups compared to DEN induced hepatic inflammation (Fig. 5). These results supported by findings of Harada et al. who reported the inhibitory effect of EPA on hepatic fibrosis by lowering hepatic Hyp content, and gene expression of collagen, and transforming growth factor -β1 in rats fed a methionine- and choline-deficient diet by immediately reducing the level of ROS [42].

We evaluated the erythrocyte membrane fatty acid fractions including ALA, AA, LA, and OA as shown in Table 1. Our results showed a significant decrease in the erythrocyte membrane ALA content accompanied with a significant increase in the erythrocyte membrane AA, LA, and OA content in DEN- induced hepatic inflammation group compared to the control group (Table 1).

**Table 1:** Cell membrane fatty acid fractions in different studied groups

**Parameters**	**ALA** (mg/ml RBCs)	**AA** (mg/ml RBCs)	**LA** (mg/ml RBCs)	**OA** (mg/ml RBCs)
**Groups**				
Control	2.24 ± 0.01	1.91 ± 0.014	1.68 ± 0.009	0.69 ± 0.023
EPA	2.28 ± 0.008	1.85 ± 0.02^a^	1.65 ± 0.024	0.67 ± 0.012
EPA-NE	2.34 ± 0.014^a^	1.83 ± 0.012^a^	1.62 ± 0.015	0.61 ± 0.021^a^
DEN	1.06 ± 0.056^a,b^	2.54 ± 0.032^a,b^	2.35 ± 0.022^a^	1.16 ± 0.027^a^
Treated I	1.55 ± 0.048^a,b^	2.37 ± 0.054^a,b^	2.08 ± 0.047^a,b^	0.93 ± 0.016^a,b^
Treated II	1.94 ± 0.077^a,b,c^	2.17 ± 0.013^a,b,c^	1.98 ± 0.074^a,b,c^	0.87 ± 0.019^a,b,c^

*P*^*a*^ value: significant difference compared to control group

*P*^*b*^ value: significant difference compared to DEN induced hepatic inflammation group

*P*^*c*^ value: significant difference compared to treated I group

In the current investigation, DEN-induced liver inflammation resulted in oxidative stress, as demonstrated by a rise in liver TBARS and a decrease in antioxidant enzyme activity (GSH and SOD), resulting in a substantial reduction in erythrocyte membrane ALA concentration. Rolo et al. proposed a link between low n-3 PUFA levels and oxidative stress, claiming that excessive reactive oxygen species formation owing to mitochondrial malfunction causes lipid peroxidation, which promotes inflammation and stellate cell activation, leading to fibrogenesis. In individuals with liver inflammation, higher levels of oxidative stress and lipid peroxidation have been found [58].

The increase in the erythrocyte membrane AA, LA, and OA content in DEN- induced hepatic inflammation group may be related to the oxidative stress and associated with inflammatory cascades observed in our study. We believe that raised AA levels are the initial stage in the progression of inflammation and eventually, cell death since the AA catabolism pathway, performed by cyclooxygenase and lipoxygenase, produces lipid pro-inflammatory mediators [59]. Consistent with this, we observed increased expression of the pro-inflammatory cytokines including IL-1β and TNF-α along with the increased level of AA (Table 1 and Fig. 5).

The cellular membranes and the capabilities we have to modify its composition and function constitute a strong weapon in the treatment of hepatic inflammation and cancer. The cell membrane and its components must be considered as important aspects in cancer treatment, and novel therapeutic techniques should be developed [4]. From this point of view, we evaluated the potential effect of EPA alone and EPA loaded silica nanoemulsion in improving the cell membrane efficiency upon administration of DEN. Data obtained showed that along with the reduction of the oxidant and inflammatory markers after administration of EPA alone or EPA loaded silica nanoemulsion, a significant increase in the erythrocyte membrane content of ALA and a significant decrease in AA, LA, and OA was observed compared to DEN induced hepatic inflammation group (Table 1and Fig. 5). It is worth to mention that there was a significant increase in the erythrocyte membrane content of ALA accompanied with a significant decrease in AA, LA, and OA in the EPA loaded silica nanoemulsion group compared to EPA only (Table 1) confirming the effectiveness of the prepared EPA in a nanoemulsion form. Besides, EPA is an omega 3 fatty acid which is characterized by its role in replacing AA in the cell membranes and gave the more elasticity and flexibility [4]. Thus in this work, EPA as an animal source of omega 3 effectively increased omega 3 fatty acids and decreased omega 6 and 9. Additionally, in EPA loaded silica nanoemulsion treated (Table 1).

In agreements with our results, Giordano and Visioli reported that moderate/appropriate amount of n-3 PUFA has an antioxidant effect. In addition, a recent study found that supplementing with LC n-3 PUFAs reduced hepatic oxidative stress and triglyceride accumulation in fatty liver caused by a high-fat diet [60]. In the current study, EPA and in particular EPA loaded silica nanoemulsion supplementation improve antioxidant status by restoring antioxidant enzyme activity and GSH levels, preventing DEN-induced hepatic oxidative damage.

Omega-3 fatty acids are promising nominees for treating a wide range of inflammatory responses which accompanies many diseases such as atherosclerosis, diabetes [61], and asthma [62]. EPA is a biologically active polyunsaturated ω-3 FA [38].

**Fig. 6:**
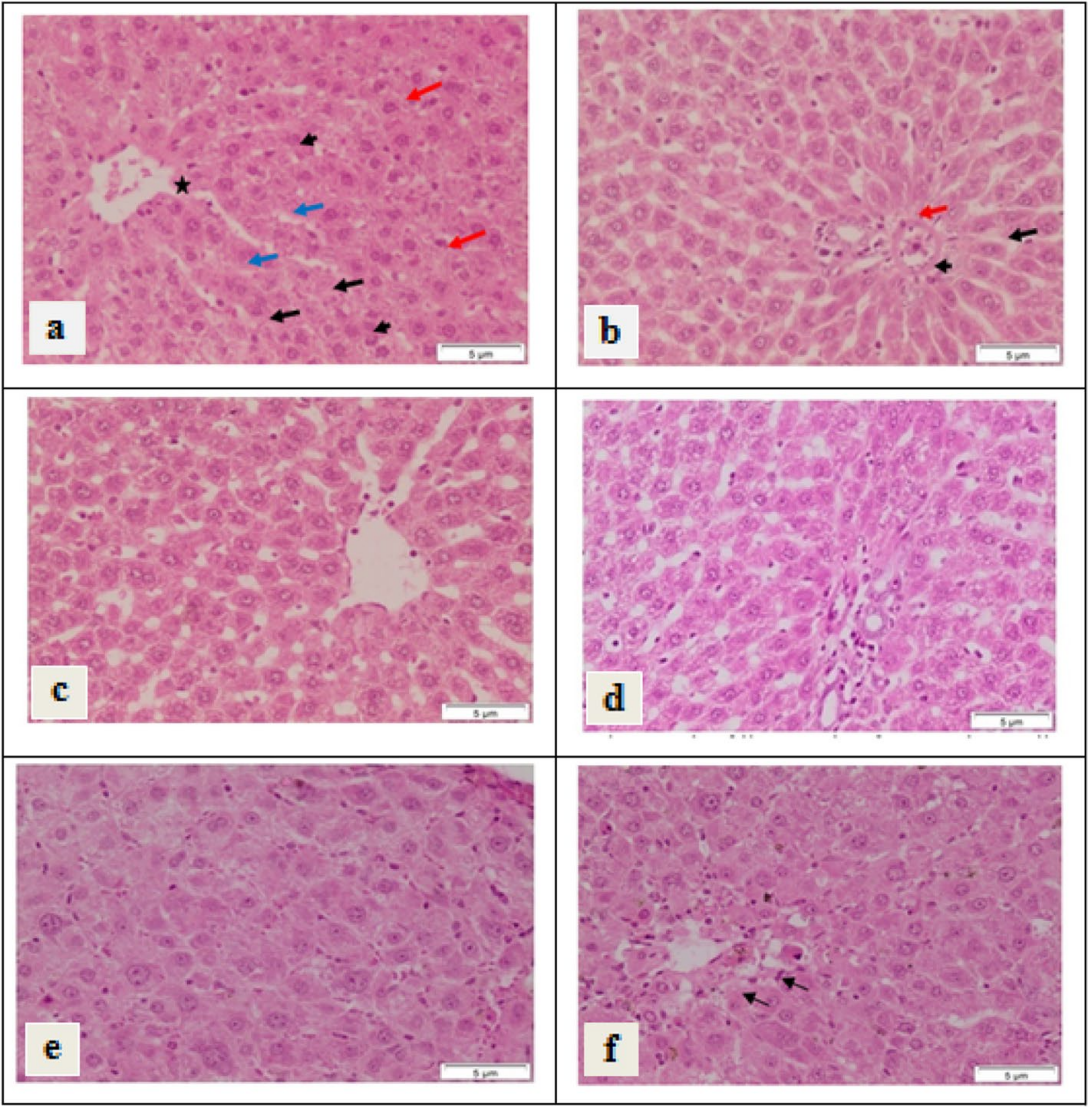
Photomicrograph of liver sections of negative control group showing **a** normal hepatocytes (arrows) radiating from the central vein (asterisk). The hepatocytes showing vesicular nuclei (arrowhead), some of it are binucleated (red arrows) and are separated with sinusoids (blue arrow). **b** Negative control group showing normal portal tract with its structures; branches of portal vein (arrow), hepatic artery (arrowhead) and bile duct (red arrow). **c** and **d** are photomicrograph of liver sections of rat that orally administered with EPA; where **c** showing normal structure of hepatocytes and sinusoids. **d** Showing normal structure of portal tract. **e** and **f** are photomicrograph of liver sections of rat that administered with EPA-NE; where **e** showing normal structure of hepatocytes and sinusoids. **f** Showing normal structure of hepatocytes and sinusoids. Some hepatocytes the surround the portal tract exhibit necrotic feature (arrows). (H&E stain, Scale bar: 5 µm)

Concomitant with the biochemical analysis, histological examination of liver sections of negative control group showed the normal structure of the hepatic lobules. The hepatocytes radiated from the central vein. It exhibited vesicular nuclei, some of it are binucleated and are separated with sinusoids (Fig. 6a). On the other hand, liver sections of negative control group showed normal portal tract with its structures; branches of portal vein, hepatic artery and bile duct (Fig. 6b).

Oral administration with EPA in a dose of 500 mg/kg/day for 4 weeks showed normal structure of hepatocytes and sinusoids (Fig. 6c) and portal tracts (Fig. 6d).

On the other hand, administration of EPA loaded silica nanoemulsion in a dose of 500 mg/kg/day for 4 weeks displayed normal structure of hepatocytes and sinusoids (Fig. 6e). Normal structure of the portal tracts was appeared. Some hepatocytes that surround the portal tract exhibited necrotic feature (Fig. 6f).

**Fig. 7:**
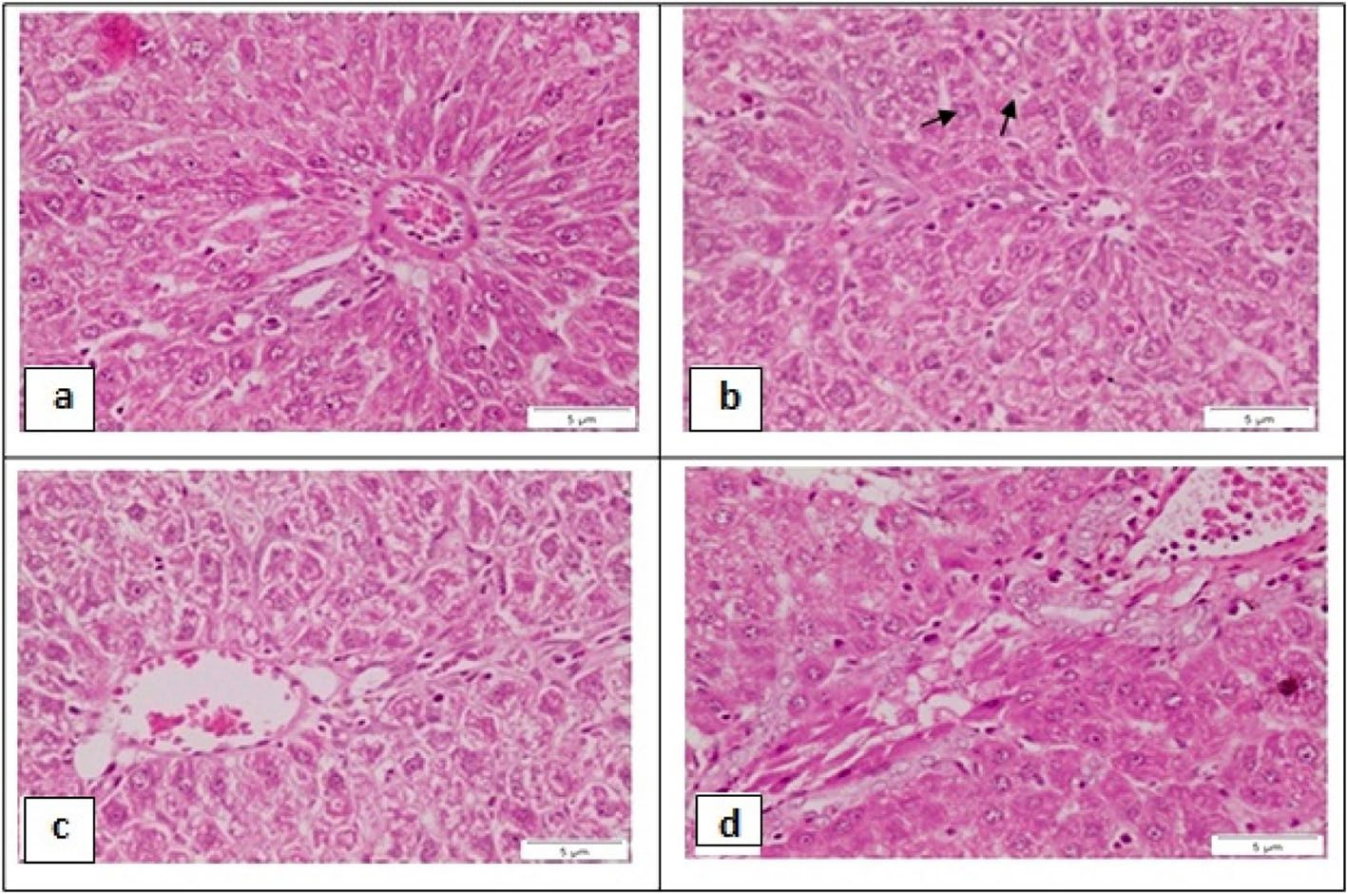
Photomicrograph of liver section from rat that administered with DEN showing: **a** loss of the normal hepatic structures, including laminae, sinusoids and dilated portal tract. **b** pseudogland formations (arrows) can be observed in high-grade macronodules. **c** Architectural pattern, along with a fibrotic stroma, lytic hepatocellular necrosis (arrowheads), no congestion was observed. **d** congestion in the portal tract that associated with necrotic feature of the surround hepatocytes. (H&E stain, Scale bar: 5 µm)

Examination of liver sections from rat that orally administered with DEN showed loss of the normal hepatic structures, including laminae, sinusoids and dilated portal tract (Fig. 7a). Formations of pseudogland can be observed in high-grade macronodules (Fig. 7b). Complete destruction of the sinusoidal architectural pattern, along with a fibrotic stroma, lytic hepatocellular necrosis, no congestion was observed (Fig. 7c). Congestion in the portal tracts that associated with necrotic feature of the surround hepatocytes were noticed (Fig. 7d).

**Fig. 8:**
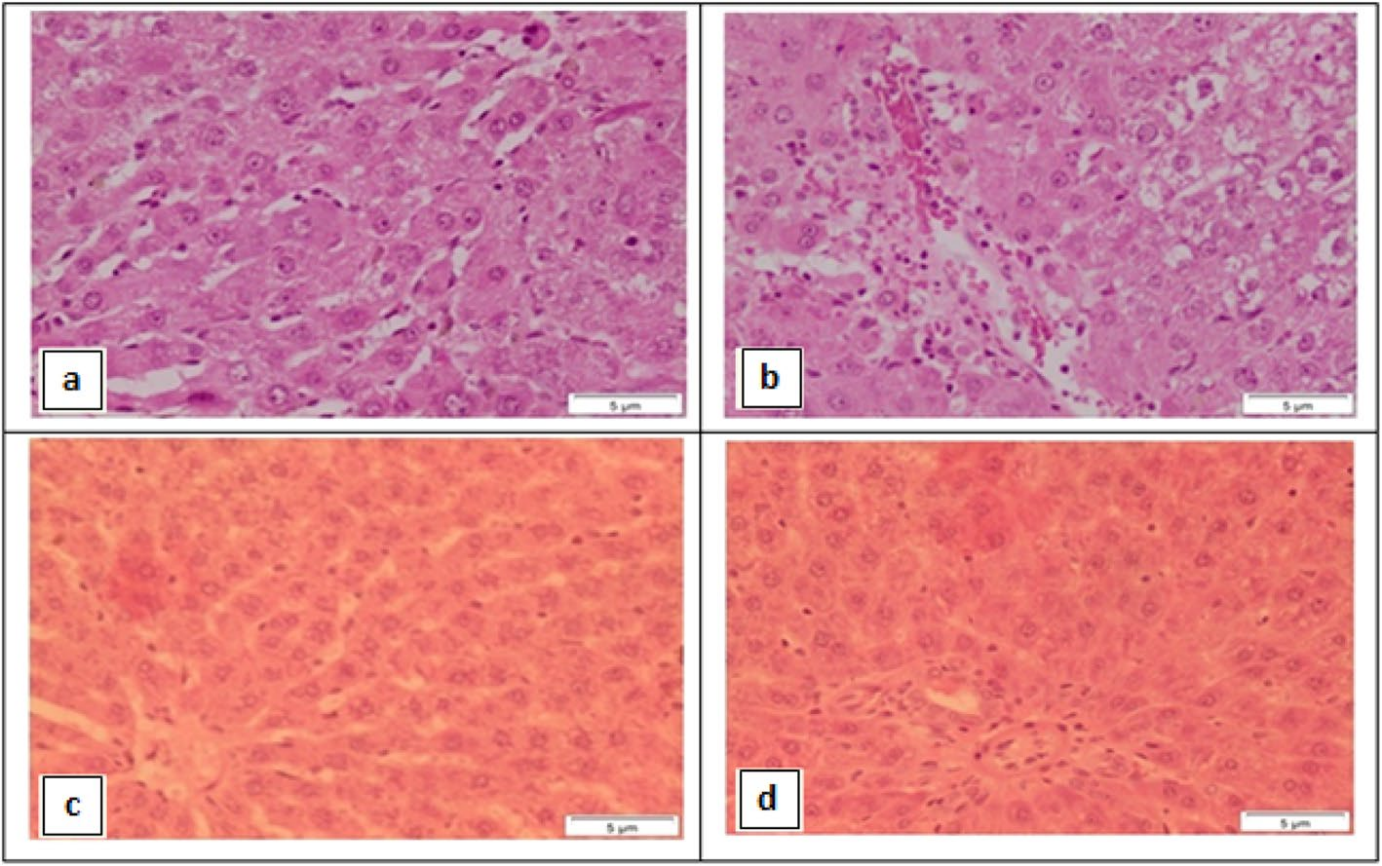
Photomicrograph of liver section showing rat that administered with DEN and in the same time treated with EPA (treated I group) showing: **a** the structure of hepatic lobule appeared more or less as normal one **b** congestion of portal tract appeared associated with mild inflammatory infiltration. Hydropic degeneration was seen (H&E stain, Scale bar: 5 µm). **c** and **d** showing rat that administered DEN and in the same time treated with EPA loaded silica nanoemulsion (treated II group). Where **c** showing the hepatic lobule appears nearly like normal control around the portal tract. **d** showing the portal tract appears nearly like normal control (H&E stain, Scale bar: 5 µm)

Histopathological examination of liver sections from rats administered DEN and in the same time treated with EPA showed the structure of hepatic lobules (Fig. 8a). In addition, Fig. 8b showed congestion of portal tracts that associated with mild inflammatory infiltration, and hydropic degeneration.

Microscopic examination of liver sections from rats that orally administered DEN and in the same time treated with EPA loaded silica nanoemulsion showed the hepatic lobules (Fig. 8c) and the portal tracts (Fig. 8d) appeared nearly like normal control.

This study clearly exhibited that oral administration of EPA alone or EPA loaded silica nanoemulsion ameliorated hepatic inflammation induced by DEN in comparison to DEN induced hepatic inflammation group in both macroscopically and microscopically examinations. Additionally, in EPA nano emulsion treated group; this effect was increased to become more or less near the control group.

We suggesting that this effect is related to the role of nanoemulsion characterized by smaller size that enhance the effect of EPA and facilitate its penetration to the cell membrane.

## Conclusion

The cellular membrane is a highly ordered and sophisticated part of the cell that is responsible for cell structure and the outside interactions. The understanding of lipid content, particularly the changes found in hepatic inflammation offers a significant potential to attenuate inflammation, oxidative stress, and reduce the risk of cancer. The anti-inflammatory and anti-oxidant effects of the EPA in a nanoemulsion form will be far better than those seen for EPA only given their excellent bio-physicochemical properties.

## Data Availability

All of the material is owned by the authors.
